# Disease patterns and causes of death of hospitalized HIV-positive adults in West Africa: a multicountry survey in the antiretroviral treatment era

**DOI:** 10.7448/IAS.17.1.18797

**Published:** 2014-04-07

**Authors:** Charlotte Lewden, Youssoufou J Drabo, Djimon M Zannou, Moussa Y Maiga, Daouda K Minta, Papa S Sow, Jocelyn Akakpo, François Dabis, Serge P Eholié, The IeDEA West Africa Collaboration

**Affiliations:** 1 Université de Bordeaux, ISPED, Centre INSERM U897, Bordeaux, France; 2 INSERM, Centre INSERM U897, Bordeaux, France; 3 Department of Internal Medicine, University Hospital Yalgado Ouédraogo, Ouagadougou, Burkina Faso; 4 Centre National Hospitalier et Universitaire Hubert Koutoukou Maga, Service de Médecine Interne, Cotonou, Benin; 5 Hôpital Gabriel-Touré, Service d'Hépatogastroentérologie, Bamako, Mali; 6 Hôpital du point G, Service de maladies infectieuses, Bamako, Mali; 7 Department of Infectious Diseases, Fann University Hospital, Dakar, Senegal; 8 Department of Infectious Diseases, Treichville University Hospital, Abidjan, Côte d'Ivoire

**Keywords:** HIV infection, Africa, hospitalization, morbidity, antiretroviral therapy, AIDS

## Abstract

**Objective:**

We aimed to describe the morbidity and mortality patterns in HIV-positive adults hospitalized in West Africa.

**Method:**

We conducted a six-month prospective multicentre survey within the IeDEA West Africa collaboration in six adult medical wards of teaching hospitals in Abidjan, Ouagadougou, Cotonou, Dakar and Bamako. From April to October 2010, all newly hospitalized HIV-positive patients were eligible. Baseline and follow-up information until hospital discharge was recorded using standardized forms. Diagnoses were reviewed by a local event validation committee using reference definitions. Factors associated with in-hospital mortality were studied with a logistic regression model.

**Results:**

Among 823 hospitalized HIV-positive adults (median age 40 years, 58% women), 24% discovered their HIV infection during the hospitalization, median CD4 count was 75/mm^3^ (IQR: 25–177) and 48% had previously received antiretroviral treatment (ART). The underlying causes of hospitalization were AIDS-defining conditions (54%), other infections (32%), other diseases (8%) and non-specific illness (6%). The most frequent diseases diagnosed were: tuberculosis (29%), pneumonia (15%), malaria (10%) and cerebral toxoplasmosis (10%). Overall, 315 (38%) patients died during hospitalization and the underlying cause of death was AIDS (63%), non-AIDS-defining infections (26%), other diseases (7%) and non-specific illness or unknown cause (4%). Among them, the most frequent fatal diseases were: tuberculosis (36%), cerebral toxoplasmosis (10%), cryptococcosis (9%) and sepsis (7%). Older age, clinical WHO stage 3 and 4, low CD4 count, and AIDS-defining infectious diagnoses were associated with hospital fatality.

**Conclusions:**

AIDS-defining conditions, primarily tuberculosis, and bacterial infections were the most frequent causes of hospitalization in HIV-positive adults in West Africa and resulted in high in-hospital fatality. Sustained efforts are needed to integrate care of these disease conditions and optimize earlier diagnosis of HIV infection and initiation of ART.

## Introduction

Since 1996, combination antiretroviral treatment (ART) has consistently improved the prognosis of HIV-positive individuals with a dramatic decrease in HIV-related morbidity and mortality, as clearly demonstrated in cohorts of seroconverters in Europe [1]. These results have also been subsequently described in Africa [2–4]. Since 2003, the number of HIV-positive individuals treated by ART increased exponentially worldwide, and in 2012, two out of three individuals eligible for ART in sub-Saharan Africa were receiving it [5]. Thus, Africa has reduced AIDS-related deaths by one-third between 2005 and 2011 [6]. Nevertheless, the case management of HIV-positive individuals remains challenging in these countries with limited resources [7]. First late diagnosis of HIV infection and late initiation of treatment lead to a high frequency of AIDS-related morbidity during the first 6–12 months of ART initiation [2,8–11]. Second, tuberculosis (TB) and invasive bacterial diseases are the most frequent reported causes of morbidity [8,9,12,13]. Their prognosis is worse when the costs of the diagnosis and treatment are born by the patient or his/her relatives [14,15]. Third, the use of poorly tolerated ART regimens may expose to frequent adverse effects and unplanned treatment discontinuations [16]. Finally, as they live longer with a longer cumulative period on ART, HIV-positive individuals may experience a wider range of co-morbidities [17], including cancers and cardiovascular diseases. The distribution of these co-morbidities may differ in Africa from those described in industrialized countries because of different environmental and pathogenic contexts and of different socio-economic and behavioural conditions. Moreover, the frequency and the type of morbidity may vary according to the immunosuppression level and duration of treatment [8].

Knowledge is limited on causes of morbidity and mortality in HIV-positive patients followed in HIV care programs in West Africa. Available data come mainly from research protocols, clinical trials or specifically designed cohort studies, which may differ from routine programs by the selection of the population and by the means engaged to diagnose clinical events. Moreover, most available data concerning hospitalizations come from retrospective studies [18–22].

Several years after the start of the ART roll-out in West Africa, a region where the ART uptake is among the lowest [6], we hypothesized that hospital wards may be the destination of most HIV-positive patients with a severe clinical event, either during regular follow-up or after an interruption of their medical follow-up.

Thus, the prospective description of HIV-positive hospitalized patients with a systematic documentation of reasons of hospitalization, diagnosis and outcome may contribute to improve the case management and plan the availability of diagnostic and therapeutic means, as well as constitute the rationale for improving preventive measures. We aimed to describe here the profile of hospitalized HIV-positive patients in the ART era in a large sample of West Africa tertiary care level hospitals, and identify the spectrum of severe illness and the cause of deaths in this context.

## Methods

### Design and settings

The International epidemiological Database to Evaluate AIDS (IeDEA) network (http://www.iedea.org/) was initiated in July 2006 to address evolving questions in HIV/AIDS research unanswerable by single cohorts by collecting and harmonizing data from multiple HIV/AIDS cohorts and programs. The IeDEA West Africa collaboration (http://mereva.net/iedea) currently involves 16 adult HIV/AIDS clinics spread over nine countries in West Africa, representing more than 60,000 HIV-positive adults followed in these centres. Within this network, we performed a six-month prospective multicentre survey from April, 2010, to October, 2010. In six HIV-specialized hospital wards in tertiary hospital in Benin, Burkina Faso, Côte d'Ivoire, Mali and Senegal, all newly hospitalized HIV-positive adults ≥18 years were eligible, if previously known as HIV-positive or newly diagnosed during the hospitalization. Baseline and follow-up information during hospitalization were recorded using a standardized clinical form.

Available investigations on site were as follows: biochemistry, haematology, blood culture, sputum microscopy and culture for *Mycobacterium tuberculosis*, drug sensitivity testing for mycobacteria, bacteriology and mycology examination of urine and cerebro-spinal fluid (CSF), hepatitis B surface (HBs) antigen, hepatitis C virus (HCV) antibody, CSF polymerase chain reaction (PCR) for herpes simplex virus (HSV) and cytomegalovirus (CMV), malaria rapid test or thick blood film, chest x-ray, ultrasound, tomodensitometry. Since their cost was borne by the patients, these investigations were not systematically performed but rather selectively prescribed.

Cotrimoxazole prophylaxis was recommended in adults with CD4 count<500 cell/mm^3^; prophylaxis for TB and *Mycobacterium avium* complex (MAC) was usually not prescribed. TB treatment (including for multidrug resistant TB) was provided free of charge by the national TB control programme. Cotrimoxazole was freely available for treatment of toxoplasmosis and sulfadiazin or clindamycin plus pyrimethamin were available in most sites at patients’ expense. Fluconazole was commonly available for the treatment of *cryptococcus* infection.

### Validation of diagnoses

Diagnoses usually relied on clinical assessment, supported by additional investigations, when performed. Within each clinic, diagnoses were validated by a local event validation committee composed of three to six specialists in internal medicine, infectious disease and in some settings gastroenterology or other speciality on request. The decisions were based on consensus. In case of disagreement, the opinion of another expert was requested. All the clinic committees used common definitions for AIDS-defining events [23], invasive infections [24,25], IRIS [26] and cancers. Diagnoses were classified as presumptive or definitive [27]. The causality assessment of side effects was adapted from the WHO-Uppsala Monitoring Centre (UMC) causality categories, by grouping the categories certain, probable and possible [28].

We provide below the definitions of the main infectious disease events (other definitions can be found elsewhere [23–25]).

The diagnosis of isolated bacteraemia or sepsis was definitive if: (1) isolation of a clinically significant pathogen from blood culture; and (2) no clinical or paraclinical focus.

The diagnosis of bacterial pneumonia was presumptive in case of: (1) consistent clinical findings; (2) chest radiographic evidence of alveolar pulmonary disease; and (3) successful response to antibiotherapy with no activity against *Pneumocystis jirovecii*; this diagnosis was definitive if: (1) consistent clinical findings, (2) chest radiographic evidence of alveolar pulmonary disease, or radiology not performed and (3) isolation of *Streptococcus pneumoniae, Haemophilus influenzae* or non-typhi Salmonella from blood culture.

The diagnosis of TB was presumptive if: (1): (a) consistent clinical findings; (b) presence of acid-fast mycobacteria on sputum sample or bronchoalveolar lavage for pulmonary TB or on normally sterile body fluid or tissue from a site other than lungs (pleural or peritoneal fluid, lymph node, liver biopsy, CSF, blood culture) for extrapulmonary TB; and (c) successful response to standard antituberculous therapy or (2): (a) consistent clinical findings >30 days; (b) no microbiological evidence of a pneumonia due to any other known pathogen for pulmonary TB or no other explanation for extrapulmonary TB; (c) unsuccessful response to standard antibiotherapy; and (d) successful response to standard antituberculous therapy.

The diagnosis of TB was definitive if: (1) consistent clinical findings; and (2) isolation of *Mycobacterium tuberculosis*, *bovis* or *africanum* from sputum sample or bronchoalveolar lavage for pulmonary TB or from at least one normally sterile body fluid or tissue from a site other than lungs (pleural or peritoneal fluid, lymph node, liver, CSF, blood culture) for extrapulmonary TB.

Diagnosis of malaria was definitive if: (1) consistent clinical findings; and (2) presence of *Plasmodium* sp on thick blood smear.

Cerebral toxoplamosis diagnosis was presumptive if: (1) recent onset of a focal neurological abnormality consistent with intracranial disease or a reduced level of consciousness; (2) brain imaging evidence of a lesion having a mass effect on computerized tomography or the radiographic appearance of which is enhanced by injection of contrast medium; and (3) successful response to therapy for toxoplasmosis.

Cryptococcosis diagnosis was definitive if: microscopy of the CSF was positive by India ink staining and/or culture on Sabouraud's medium and/or cryptococcal antigen was detected by latex agglutination.

### Descriptive analysis

We described first the reasons of admission according to the symptomatology that justified entry in hospital. The reasons of hospitalization were then categorized based on the diagnoses of the disease conditions considered to have led to hospitalization. If a patient had multiple diagnoses during one hospitalization, we determined one underlying cause of hospitalization by applying the following priority order: (1) WHO stage 4 opportunistic disease; (2) WHO stage 3 opportunistic disease; (3) other infection; (4) other cancer; (5) WHO stage 4 cachexia; (6) other disease; and (7) non-specific event including symptomatic diagnosis and non-specific WHO stage 3 events (weight loss >10%, chronic diarrhoea, persistent fever). We then grouped the underlying causes of hospitalization into four broad categories: AIDS, other infection, other diseases and non-specific diseases and described the characteristics of the hospitalized patients accordingly. Finally, we described the overall numbers and percentages of all diseases diagnosed during hospitalization as well as the fatal hospitalizations. We considered as the underlying cause of death the disease or injury, which initiated the train of morbid events leading to death [29].

### Statistical analysis

Categorical variables were described using frequencies and percentage. Continuous variables were described using median and interquartile range (IQR).

A logistic regression model was used to analyze the association between in-hospital mortality and the following variables: gender, age, duration since HIV diagnosis, history of cotrimoxazole prophylaxis, history of ART, WHO clinical stage, body mass index, haemoglobinemia, CD4 cell count and underlying cause of hospitalization. Variables with a *p*<0.25 in univariate analysis were included in multivariate analysis. Analyses were performed with SAS^®^ software, version 9.1.3 (SAS institute Inc. Cary, North Caroline, USA).

### Ethical considerations

The IeDEA West Africa collaboration obtained the authorization from the Ethics Committee “Comité de Protection des Personnes Sud-Ouest et Outre-Mer III” in Bordeaux, France, and each site obtained the authorization from its National Ethics Committee.

## Results

### Patients’ characteristics

From April to September 2010, 824 hospitalizations were recorded in 823 HIV-positive adults in six participating tertiary hospitals in five countries in West Africa. In five out of the six wards where this figure was available, the coverage of HIV screening among hospitalized patients ranged between 98 and 100% during the study period; 58% of these patients were women and the median age at admission was 40 years (IQR: 33–48) (Table 1). Overall, 24% of these patients discovered that they were HIV-positive during this hospitalization, 94% were at WHO stage 3 or 4 at hospital admission and median CD4 count was 75/mm^3^ (IQR: 25–177). Among those who knew their HIV-positive status before the hospitalization (*N*=623), the median time elapsed since HIV diagnosis was 3.7 months (IQR: 0.6–31.7), 63% were already on ART and 68% had initiated cotrimoxazole prophylaxis. Among the 392 patients who had ever received ART before hospital entry, the median CD4 count at ART initiation was 90/mm^3^ (IQR: 32–178) and the median CD4 count closest to hospital admission was 93/mm^3^ (IQR: 30–231). The median CD4 count of the 431 patients not on ART at hospital entry was 53/mm^3^ (IQR: 18–127); 39 (9%) of these individuals initiated ART during hospitalization.

**Table 1:** Characteristics of HIV-positive adults newly hospitalized from April to September 2010, overall and according to the underlying cause of hospitalization, IeDEA West Africa Collaboration (*N*=823)

	All causes	AIDS	Other infections	Other diseases	Non-specific illness^a^
	
	(*n*=823)	(*n*=445, 54.0%)	(*n*=268, 32.5%)	(*n*=62, 7.5%)	(*n*=49, 6.0%)
Median age (years) (interquartile range)	40 (33–48)	39 (34–47)	40 (32–47)	40 (34–53)	40 (34–47)
Women (%)	58	56	62	61	55
HIV type (%)					
1	94	94	93	98	92
2	4	4	5	0	6
1 +2	2	2	2	2	2
Diagnostic of HIV (%)					
During hospitalization	24	25	25	11	20
<6 months before hospitalization	42	48	36	29	35
≥6 months before hospitalization	34	27	39	60	45
Known duration of HIV infection if HIV diagnosed before hospitalization (*n*=623) (months) (interquartile range)	3.7 (0.6–31.7)	2.1 (0.5–17.8)	7.6 (0.7–38.8)	18.6 (1.7–52.5)	10.3 (1.2–39.6)
WHO stage at entry (%)					
4	54	80	23	35	12
3	40	19	66	48	80
2 or 1	6	1	11	17	8
Median body mass index (kg/m^2^) (interquartile range)	16.8 (14.5–19.6)	16.5 (14.3–19.1)	16.9 (14.2–19.6)	18.3 (16.1–21.4)	17.7 (14.5–21.5)
Median haemoglobinemia (g/l) (interquartile range)	9.0 (7.0–10.0)	8.0 (7.0–10.0)	8.0 (6.0–10.0)	10.0 (8.0–11.0)	8.0 (6.0–10.0)
Median last CD4+cell count^b^ (*n*=625) (/mm^3^) (interquartile range)	75 (25–177)	58 (19–128)	88 (28–231)	154 (46–333)	106 (54–193)
History of cotrimoxazole prophylaxis (%)	52	49	51	65	60
History of ART use (%)	48	45	45	71	59
Median time since first ART (*n*=388) (months) (interquartile range)	13.2 (2.0–44.5)	5.1 (1.3–31.1)	25.2 (3.3–57.2)	30.4 (2.9–56.6)	14.7 (3.6–38.7)
Median CD4+cell count at ART initiation (*n*=300) (/mm^3^) (interquartile range)	90 (32–176)	84 (29–165)	90 (32–198)	118 (61–173)	97 (50–193)
Median duration of hospitalization (days) (interquartile range)	13 (7–22)	15 (8–27)	11 (7–19)	13 (6–20)	9 (6–12)
Death in hospital (*n*=315) (%)	38	46	34	23	16

HIV: human immunodeficiency virus; ART: combination antiretroviral treatment; WHO: World Health Organization.

a Non-specific diseases: symptomatic diagnosis, non-specific WHO stage 3 events (weight loss>10%, chronic diarrhoea, persistent fever);

b median time between date of hospitalization and last CD4 count: two days (IQR: −21 to +7).

### Causes of hospitalization and diseases diagnosed

The most frequent reason for admission was a non-specific morbidity event (88%). The most frequent underlying cause of hospitalization was an AIDS-defining condition (54%), followed by non-AIDS-defining infections (32%), other diseases (8%) and non-specific illnesses (6%). This distribution did not differ between men and women. For patients who knew their HIV infection for more than six months (*N*=283), the most frequent underlying cause of hospitalization remained AIDS (42%), followed by non-AIDS-defining infections (37%), other diseases (13%) and non-specific illnesses (8%). Overall, the most frequent diseases diagnosed were: tuberculosis (29%), pneumonia (15%), malaria (10%) and toxoplasmosis (10%) (Tables 2–6). TB was diagnosed in 242 patients overall including 151 cases of extrapulmonary TB (62%). IRIS was diagnosed in 45 (5.5%) patients, of whom 27 occurred with TB and nine with cryptococcosis (Tables 2–4).

**Table 2:** Distribution of fungal and parasitic diseases among HIV-positive adults newly hospitalized from April to September 2010, IeDEA West Africa Collaboration

		Classification, *N* (%)^a^			Diagnosis, *N*			
					
	*N* (%)^a^	AIDS	WHO stage 4	WHO stage 3	Presumptive	Definitive	Suspected IRIS, *N*	Suspected Adverse effect, *N*
Total number of patients	824 (100)	445 (100)	388 (100)	232 (100)			45	45
Fungal diseases								
Oesophageal candidiasis	25 (3.0)	25 (5.6)	25 (6.4)		11	14	1	
Oral candidiasis	55 (6.7)			47 (20.2)	28	27		
Digestive or vaginal candidiasis	5 (0.6)				5	–		
Extrapulmonary cryptococcosis	49 (5.9)	49 (10.9)	49 (12.5)		3	46	9	
Pulmonary pneumocystosis	7 (0.8)	7 (1.6)	7 (1.8)		7	–		
Parasitic diseases								
Amebiasis^b^	3 (0.4)				–	3		
Anguillulosis	2 (0.2)				–	2		
Ankylostomiasis	2 (0.2)				–	2		
Ascabiosis	1 (0.1)				–	1		
Bilharziosis urinary	1 (0.1)				–	1		
Blastocystosis	1 (0.1)				–	1		
Cryptosporidiosis	11 (1.3)	11 (2.5)	11 (2.8)		–	11	1	
Giardiasis	6 (0.7)				–	6		
Isosporiasis	16 (1.9)	16 (3.6)	16 (4.1)		6	10		
Leishmaniosis cutaneous	1 (0.1)				–	1		
Malaria^c^	82 (10.0)				8	74		2
Microsporidiosis	6 (0.7)				–	6		
Cerebral toxoplasmosis	78 (9.5)	78 (17.4)	78 (19.9)		78	–	2	

a Several illnesses may have occurred during the hospitalization of a given patient;

b amebiasis: digestive (*n*=2), liver abscess (*n*=1);

c malaria: severe *Plasmodium falciparum* infection (*n*=26 of including two cases of blackwater fever).

**Table 3:** Distribution of mycobacterial, bacterial and other invasive infectious diseases among HIV-positive adults newly hospitalized from April to September 2010, IeDEA West Africa Collaboration

		Classification, *N* (%)^a^			Diagnosis, *N*			
					
	*N* (%)^a^	AIDS	WHO stage 4	WHO stage 3	Presumptive	Definitive	Suspected IRIS, *N*	Suspected Adverse effect, *N*
Total number of patients	824 (100)	445 (100)	388 (100)	232 (100)			45	45
Mycobacterial diseases								
Tuberculosis	242 (29.4)	242 (54.0)	151 (38.6)	91 (39.1)	167	75	21	
Atypical mycobacteriae^b^	5 (0.6)	5 (1.1)	5 (1.3)		5	–	5	
Bacterial/ invasive infectious diseases								
Infectious diarrhoea^c^	69 (8.4)				68	1	1	
*E. coli* enteritis	11 (1.3)			11 (4.7)	–	11		
Salmonella non-typhi	8 (1.0)	3 (0.7)	3 (0.8)	4 (1.7)	1	7		
Shigellosis diarrhoea	1 (0.1)			1 (0.4)	–	1		
Pneumonia	124 (15.0)	8 (1.8)	8 (2.0)	116 (50.0)	111	13		
Lung abscess	2 (0.2)				–	2		
Bacterial pleurisy	4 (0.5)			4 (1.7)	1	3		
Endocarditis	2 (0.2)			2 (0.9)	1	1		
Meningitis, meningo-encephalitis	37 (4.5)			35 (15.1)	29	8	1	
Pyelonephritis	11 (1.3)			11 (4.7)	7	4		
Low urinary tract infection	73 (8.9)				3	70		
Salpingitis	1 (0.1)			1 (0.4)	–	1		
Osteomyelitis	1 (0.1)			1 (0.4)	–	1		
Myositis	1 (0.1)			1 (0.4)	–	1		
Otitis	11 (1.3)				2	9		
Sinusitis	5 (0.6)				–	5		
Erysipelas	1 (0.1)			1 (0.4)	1	–		
Dermo-hypodermitis	8 (1.0)				2	6		
Tetanus	4 (0.5)				–	4		
Sepsis	69 (8.4)			69 (29.6)	33	26		

a Several illnesses may have occurred during one hospitalization in a given patient;

b type of mycobacteriae non-specified;

c infectious diarrhoea: germ not identified.

**Table 4:** Distribution of viral diseases and neoplasia among HIV-positive adults newly hospitalized from April to September 2010, IeDEA West Africa Collaboration

		Classification, *N* (%)^a^			Diagnosis, *N*			
								Suspected
	*N* (%)^a^	AIDS	WHO stage 4	WHO stage 3	Presumptive	Definitive	Suspected IRIS, *N*	Adverse effect, *N*
Total number of patients	824 (100)	445 (100)	388 (100)	232 (100)			45	45
Viral diseases								
CMV infection^b^	3 (0.4)	3 (0.7)	3 (0.8)		–	3	1	
Herpes infection^c^	10 (1.1)	5 (1.1)	5 (1.3)		10	–		
HIV encephalopathy	19 (2.3)	19 (4.2)	19 (4.9)		16	3		
Molluscum contagiosum	3 (0.4)				1	2		
PML	1 (0.1)	1 (0.2)	1 (0.3)		1	–		
VZV infection	1 (0.1)				–	1		
Viral hepatitis B	17 (2.1)				–	17		
Tumour								
Invasive cervix carcinoma	2 (0.2)	2 (0.4)	2 (0.5)		–	2		
Kaposi sarcoma	21 (2.5)	21 (4.7)	21 (5.4)		10	11	3	
NHL	8 (1.0)	8 (1.8)	8 (2.0)		5	3		
Hepatocarcinoma	4 (0.5)				3	1		
Other tumors^d^	6 (0.7)				1	5		
In situ cervical intraepithelial neoplasis	4 (0.5)				–	4		
Benign tumour or tumour of unspecified evolution	7 (0.8)				3	4		

CMV: cytomegalovirus; PML: progressive multifocal encephalopathy; VZV: varicella zoster virus; NHL: non-Hodgkin lymphoma.

a Several illnesses may have occurred during the hospitalization of a given patient;

b CMV infection: localization non-specified;

c herpes infection: genital (*n*=4), oral (*n*=2), anal (*n*=2), meningitis (*n*=1), cutaneous (*n*=1), digestive (*n*=1);

d other cancers: multiple myeloma (*n*=1), pancreas cancer (*n*=1), ano-rectal cancer (*n*=1), abdominal tumour (*n*=1), ENT cancer (*n*=1), epidermoid carcinoma of the eye (*n*=1).

The proportion of malaria cases was 7.8% in patients using a cotrimoxazole prophylaxis prior to hospitalization as compared to 10% in patients without prophylaxis (*p*=0.05). The distribution of other infections did not differ between these two groups.

Adverse effects of treatment were suspected in 45 patients (5.5%), anaemia (*n*=19) and cutaneous effects (*n*=8) being the most frequent ones (Tables 2 and 5, and 6). Adverse events were the underlying cause of hospitalization in 16 cases (2% of the total number of hospitalizations).

**Table 5:** Distribution of other diseases among HIV-positive adults newly hospitalized from April to September 2010, IeDEA West Africa Collaboration

		Classification, *N* (%)^a^			Diagnosis, *N*			
					
	*N* (%)^a^	AIDS	WHO stage 4	WHO stage 3	Presumptive	Definitive	Suspected IRIS, *N*	Suspected Adverse effect, *N*
Total number of patients	824 (100)	445 (100)	388 (100)	232 (100)			45	45
Other diseases								
Digestive^b^	37 (4.5)				5	32		1
Bronchopulmonary^c^	2 (0.2)				1	1		
Cardiovascular^d^	53 (6.4)				15	37		3
Neurologic^e^	28 (3.4)				24	4		4
Psychiatric^f^	9 (1.1)				4	5		1
Renal^g^	41 (5.0)		12 (3.1)		11	30		2
Genital	11 (1.3)				2	9		
Osteo-articular	5 (0.6)				2	3		
Cutaneous	14 (1.7)				3	11		8^i^
Endocrine^h^	6 (0.7)				1	5		
Ocular	1 (0.1)				–	1		
Trauma	1 (0.1)				1	–		

a Several illnesses may have occurred during the hospitalization of a given patient;

b digestive: gastroduodenal ulcer (*n*=15), gastritis (*n*=3), oesophagitis (*n*=4), duodenogastric reflux (*n*=3), appendicitis/peritonitis (*n*=3), cholecystitis (*n*=6), calculus of gallbladder (*n*=2), digestive haemorragiae (*n*=1), functional intestinal disorder (*n*=7), anal fistula (*n*=1);

c bronchopulmonary: pleuresia (*n*=1), pneumothorax (*n*=1);

d cardiovascular: stroke (*n*=19), heart failure (*n*=11), pericarditis (*n*=7), high blood pressure (*n*=6), peripheral venous disease (*n*=3), pulmonary embolism (*n*=2);

e neurologic: peripheral neuropathy [*n*=12 including four suspected adverse effects of zidovudine (*n*=1), d4T (*n*=3)], encephalitis (*n*=8), cerebral abscess (*n*=7), Guillain-Barre syndrome (*n*=1);

f psychiatric: delirium ascribed to efavirenz (*n*=1);

g renal: renal insufficiency (*n*=24 including two suspected adverse effect of tenofovir), HIV associated nephropathy (*n*=12), glomerular disease (*n*=4), ureteral lithiasis (*n*=1);

h endocrine: diabetes (*n*=4), thyroid, multinodular goitre (*n*=1), surrenal insufficiency (*n*=1);

i toxidermia (*n*=8 including four cases ascribed to nevirapine and one to isoniazid).

**Table 6:** Distribution of non-specific diseases among HIV-positive adults newly hospitalized from April to September 2010, IeDEA West Africa Collaboration

		Classification, *N* (%)^a^			Diagnosis, *N*			
					
	*N* (%)^a^	AIDS	WHO stage 4	WHO stage 3	Presumptive	Definitive	Suspected IRIS, *N*	Suspected Adverse effect, *N*
Total number of patients	824 (100)	445 (100)	388 (100)	232 (100)			45	45
Non-specific diseases								
Cachexia	7 (0.8)	7 (1.6)	7 (1.8)		1	6		
Fever	3 (0.4)			2 (0.9)	1	2		
Diarrhoea	32 (3.8)			26 (11.2)	24	8		
Anaemia	123 (15.0)				5	118		19^b^
Thrombopenia	2 (0.2)				1	1		
Pancytopenia	16 (1.9)				0	16		3^c^
Toxic hepatitis	6 (0.7)				5	1		1
Metabolic disorder	1 (0.1)				0	1		1
Electrolytic disorder	5 (0.6)				0	5		
Dehydration/hypovolemic shock	8 (1.0)				4	4		
Denutrition	9 (1.1)				2	7		
Altered consciousness	1 (0.1)				1	0		
Lymphadenopathy	2 (0.2)				0	2		

a Several illnesses may have occurred during the hospitalization of a given patient;

b 16/19 anaemia ascribed to zidovudine;

c 2/3 pancytopenia ascribed to zidovudine.

Anaemia was common during hospitalization (*n*=123, 15%), mostly associated with another diagnosis and in a few instances (*n*=15, 2%), it was the underlying cause of hospitalization.

### Characteristics of patients according to the underlying cause of hospitalization

Median CD4 count was lower in patients with an AIDS diagnosis (58/mm^3^) or another infection (88/mm^3^) than in patients with other diseases (154/mm^3^) (Table 1).

The diagnosis of HIV infection was revealed during the hospitalization or within the preceding six months in 73% of patients with an AIDS diagnosis.

### Hospitalization outcome

The median length of stay in hospital was 13 days (IQR: 7–22). Overall, 315 (38%) patients died during their hospitalization and the median hospital stay prior to death was nine days (IQR: 3–20). Case fatality rate was 34% in patients on ART prior to hospitalization, 40% in patients without ART but on cotrimoxazole and 43% in patients with neither ART nor cotrimoxazole. It was 46% among patients who were hospitalized because of an AIDS-defining event.

In multivariate analysis, the following variables were associated with in-hospital mortality: older age (*p*=0.02); WHO clinical stage 3 (*p*=0.003) and 4 (*p*=0.002) versus stage 1 or 2; CD4 count <50 (*p*<0.0001), 50–200 (*p*=0.02) and unknown (*p*<0.0001) versus CD4 count >200/mm^3^; AIDS as the underlying cause of hospitalization (*p*=0.0005) and non-AIDS-defining infections (*p*=0.01) versus non-specific illnesses.

The underlying cause of death was AIDS (63%), non-AIDS-defining infections (26%), other diseases (7%) and non-specific illnesses or of unknown cause (4%). This distribution did not differ between men and women. The 198 patients who died from AIDS presented the following AIDS-defining diseases: TB (*n*=113, 35.9% of deaths), cerebral toxoplasmosis (*n*=31, 9.8%), cryptococcosis (*n*=29, 9.2%), HIV encephalopathy (*n*=13, 4.1%), Kaposi sarcoma (*n*=9, 2.9%), isosporidiosis (*n*=6, 1.9%), oesophageal candidiasis (*n*=6, 1.9%), pneumocystosis (*n*=5, 1.6%), non-Hodgkin lymphoma (*n*=5, 1.6%), cryptosporidiosis (*n*=4, 1.3%), cachexia (*n*=3, 1.0%), CMV infection (*n*=2, 1.0%), herpes infection (*n*=1, 0.3%) and recurrent bacterial pneumonia (*n*=1, 0.3%). The most frequent fatal non-AIDS-defining infections were sepsis (*n*=21, 6.7% of deaths), malaria (*n*=11, 3.5%), meningitis (*n*=9, 2.9%) and infectious diarrhoea (*n*=9, 2.9%).

## Discussion

In 2010, seven years after ART started to be scaled-up in Africa, AIDS and other infections remain the first cause of hospitalization in HIV-positive adults in these tertiary care hospitals throughout West Africa. The most frequent diseases diagnosed were TB, pneumonia, malaria and toxoplasmosis. HIV infection was diagnosed during hospitalization or in the previous six months in the majority of the patients and half of them had already received ART before hospitalization. More than one-third of the patients died during their hospitalization and the most frequent fatal diseases were TB, cerebral toxoplasmosis, cryptococcosis and sepsis.

We provided a thorough description of a large sample of hospitalized patients in five countries in West Africa, and all patients hospitalized in participating wards during the survey period were included with an almost systematic HIV screening procedure.

We conducted this survey in reference hospitals where diagnostics tools are generally available and likely to be used, despite a general context of scarce resources. Local teams according to standardized definitions validated diagnoses. Nevertheless, some diseases may have not been properly diagnosed due to insufficient or incomplete investigation. Indeed the cost of most investigations remained born by the patient. For instance, some meningitis or sepsis may have been partially characterized. However, symptomatic diagnoses remained rare in these reference settings. Conversely, conducting the survey in tertiary hospitals may have selected the patients with the most severe illnesses. Since we hypothesized that hospital wards may be the destination of most HIV-positive patients with a severe clinical event, we believe that, apart from death outside hospital, we identified the most reachable severe conditions whose prevention, screening, diagnosis and case management need to be improved in priority.

Indeed, another limitation is the lack of identification of patients with the most severe disease conditions and who died before reaching the hospital. This happens often by lack of financial means and is a common drawback of HIV case management in sub-Saharan Africa. Finally, we acknowledge the possibility of missing patients hospitalized in other wards where HIV screening may have been omitted whereas the HIV screening coverage was extremely high in the participating wards.

HIV infection was frequently diagnosed during hospitalization or shortly before. The median CD4 at the time of hospital admission was quite low and time on ART was short, as previously reported in South Africa [30]. The short time interval between ART initiation and hospitalization was clearly the reflection of late initiation of ART with its consequences of high incidence of morbidity and mortality in the first months on treatment [9,31]. As in this series, CD4 count at ART initiation remains too often below 200/mm^3^ in resource-limited settings even in the recent years [32].

The high fatality we observed among these hospitalized patients and the factors associated with in-hospital mortality have already been described in other African settings [21,33] and may be the consequence of several parameters: the late diagnosis of HIV infection (during hospitalization or less than six months before in the majority of patients in our study) [34] associated with a low CD4 count [35]; the severity of diseases at hospital entry that may be due in part to late presentation; and finally the limited means available for intensive care. TB, cerebral toxoplasmosis and cryptococcosis were the more frequent diseases diagnosed in the patients who died in our study, in agreement with the limited number of autopsy studies performed throughout sub-Saharan Africa [36].

TB and pneumonia were frequent causes of hospitalization as already reported in HIV-positive individuals throughout sub-Saharan Africa [21,33,37,38]. These diseases remain common within a wide range of CD4 counts [39] and are still the most frequent causes of death in this population [40]. Indeed, almost half of the patients with a diagnosis of TB died during their hospitalization. The important contribution of TB as a cause of death in HIV-positive adults in Africa has been previously described [9,41,42]. Earlier diagnosis of HIV infection and initiation of ART, but also intensified TB case finding, with enhanced diagnosis tools and systematic linkage between TB and HIV services are necessary to expect a decrease of such TB-related mortality [43]. In West Africa, isoniazid prophylaxis is not commonly prescribed and further research is needed to identify the reasons of this discrepancy between recommendations and practice. The high fatality of cryptococcosis even since the availability of ART was confirmed in our series (59%) [44–46]. Since diagnosis and treatment of cryptococcosis remain difficult to implement in resource-limited settings, earlier ART initiation remains the best way to prevent death with cryptococcosis, as recommended by WHO [47]. In addition, implementation of point-of-care cryptococcal antigen tests could also be very helpful as well as the first-line treatment option with amphotericin B [47,48]. More generally, the improvement in diagnostic tools and the availability of a whole range of free of charge treatment options are needed to optimize treatment strategies, guarantee treatment adherence and thus lower the burden of opportunistic infections and their related case fatality [49].

In our study, malaria was diagnosed in 10% of hospitalized patients. Occurrence of malaria is common in HIV-positive individuals in West Africa and parasitemia increases while CD4 count decreases [50]. However, the fatality was lower than for other morbid conditions, partly due to the wide use of cotrimoxazole prophylaxis.

Adverse effects of treatment were not frequently suspected as causes of hospitalization. In fact most adverse effects of treatment in HIV-positive patients are of low or moderate grade and do not require hospitalization [51].

In contrast with high-resource settings [52,53], the decrease in overall mortality among HIV-positive individuals in sub-Saharan Africa due to the widespread use of ART has not yet resulted, at least in our sample, in a shift toward chronic non-communicable diseases as causes of hospitalization. This is related to the late initiation of ART that cannot prevent occurrence of AIDS-related complications and undermines its effectiveness. In fact, even in individuals with a good response to treatment, the mortality risk remains high if they experienced an AIDS event before ART initiation [54].

## Conclusions

AIDS, mostly TB-related, remained the primary cause of hospitalization of HIV-positive adults in 2010 in tertiary care hospitals throughout West Africa; bacterial diseases were also frequent causes of hospitalization. In-hospital fatality remained high also. Sustained efforts are needed to generalize earlier diagnosis of HIV infection together with earlier initiation of ART as clearly recommended by international guidelines.

## Appendix

The IeDEA West Africa Collaboration Study Group (as of 2013):

**Executive Committee:** François Dabis (Principal Investigator, Bordeaux, France), Emmanuel Bissagnene (Co-Principal Investigator, Abidjan, Côte d'Ivoire), Elise Arrivé (Bordeaux, France), Patrick Coffie (Abidjan, Côte d'Ivoire), Didier Ekouevi (Abidjan, Côte d'Ivoire), Antoine Jaquet (Bordeaux, France), Valériane Leroy (Bordeaux, France), Charlotte Lewden (Bordeaux, France), Annie J Sasco (Bordeaux, France).

**Participating sites (*members of the Steering Committee):**

Benin, Cotonou:

Adults: Djimon Marcel Zannou*, Carin Ahouada, Jocelyn Akakpo, Christelle Ahomadegbé, Jules Bashi, Alice Gougounon-Houéto, Angèle Azon-Kouanou, Fabien Houngbé, Jean Sehonou (CNHU Hubert Maga).

Pediatrics: Sikiratou Koumakpaï*, Florence Alihonou, Marcelline d'Almeida, Irvine Hodonou, Ghislaine Hounhoui, Gracien Sagbo, Leïla Tossa-Bagnan, Herman Adjide (CNHU Hubert Maga).

Burkina Faso:

Adults: Joseph Drabo*, René Bognounou, Arnaud Dienderé, Eliezer Traore, Lassane Zoungrana, Béatrice Zerbo (CHU Yalgado, Ouagadougou), Adrien Bruno Sawadogo*, Jacques Zoungrana, Arsène Héma, Ibrahim Soré, Guillaume Bado, Achille Tapsoba (CHU Souro Sanou, Bobo Dioulasso)

Pediatrics: Diarra Yé*, Fla Kouéta, Sylvie Ouedraogo, Rasmata Ouédraogo, William Hiembo, Mady Gansonré (CH Charles de Gaulle, Ouagadougou).

Côte d'Ivoire, Abidjan:

Adults: Eugène Messou*, Joachim Charles Gnokoro, Mamadou Koné, Guillaume Martial Kouakou, (ACONDA-CePReF); Clarisse Amani Bosse*, Kouakou Brou, Achi Isidore Assi (ACONDA-MTCT-Plus); Henri Chenal*, Denise Hawerlander, Franck Soppi (CIRBA); Albert Minga*, Yao Abo, Jean-Michel Yoboue (CMSDS/CNTS); Serge Paul Eholié*, Mensah Deborah Noelly Amego, Viviane Andavi, Zelica Diallo, Frédéric Ello, Aristophane Koffi Tanon (SMIT, CHU de Treichville), Serge Olivier Koule*, Koffi Charles Anzan, Calixte Guehi (USAC, CHU de Treichville);.

Pediatrics: Edmond Addi Aka*, Koffi Ladji Issouf, Jean-Claude Kouakou, Marie-Sylvie N'Gbeche, (ACONDA-CePReF); Touré Pety*, Divine Avit-Edi (ACONDA-MTCT-Plus); Kouadio Kouakou*, Magloire Moh, Valérie Andoblé Yao (CIRBA); Madeleine Amorissani Folquet*, Marie-Evelyne Dainguy, Cyrille Kouakou, Véronique Tanoh Méa-Assande, Gladys Oka-Berete, Nathalie Zobo, Patrick Acquah, Marie-Berthe Kokora (CHU Cocody); Tanoh François Eboua*, Marguerite Timité-Konan, Lucrèce Diecket Ahoussou, Julie Kebé Assouan, Mabéa Flora Sami, Clémence Kouadio (CHU Yopougon).

Ghana, Accra:

Pediatrics: Lorna Renner*, Bamenla Goka, Jennifer Welbeck, Adziri Sackey, Seth Ntiri Owiafe (Korle Bu Teaching Hospital).

Guinea-Bissau:

Adults: Christian Wejse*, Zacarias José Da Silva*, Joao Paulo (Bandim Health Project), The Bissau HIV cohort study group: Amabelia Rodrigues (Bandim Health Project), David da Silva (National HIV program Bissau), Candida Medina (Hospital National Simao Mendes, Bissau), Ines Oliviera-Souto (Bandim Health Project), Lars Østergaard (Dept. of Infectious Diseases, Aarhus University Hospital), Alex Laursen (Dept. of Infectious Diseases, Aarhus University Hospital), Morten Sodemann (Dept. of Infectious Diseases, Odense University Hospital), Peter Aaby (Bandim Health Project), Anders Fomsgaard (Dept. of Virology, Statens Serum Institut, Copenhagen), Christian Erikstrup (Dept. of Clinical Immunology), Jesper Eugen-Olsen (Dept. of Infectious Diseases, Hvidovre Hospital, Copenhagen).

Mali, Bamako:

Adults: Moussa Y Maïga*, Fatoumata Fofana Diakité, Abdoulaye Kalle, Drissa Katile (CH Gabriel Toure), Hamar Alassane Traore*, Daouda Minta*, Tidiani Cissé, Mamadou Dembelé, Mohammed Doumbia, Mahamadou Fomba, Assétou Soukho Kaya, Abdoulaye M Traoré, Hamady Traoré, Amadou Abathina Toure (CH Point G).

Pediatrics: Fatoumata Dicko*, Mariam Sylla, Alima Berthé, Hadizatou Coulibaly Traoré, Anta Koïta, Niaboula Koné, Clémentine N'Diaye, Safiatou Touré Coulibaly, Mamadou Traoré, Naïchata Traoré (CH Gabriel Toure).

Nigeria:

Adults: Man Charurat* (UMB/IHV), Samuel Ajayi*, Georgina Alim, Stephen Dapiap, Otu (UATH, Abuja), Festus Igbinoba (National Hospital Abuja), Okwara Benson*, Clément Adebamowo*, Jesse James, Obaseki, Philip Osakede (UBTH, Benin City), John Olasode (OATH, Ile-Ife).

Senegal, Dakar:

Adults: Moussa Seydi*, Papa Salif Sow, Bernard Diop, Noël Magloire Manga, Judicael Malick Tine, Coumba Cissé Bassabi (SMIT, CHU Fann),

Pediatrics: Haby Signate Sy*, Abou Ba, Aida Diagne, Hélène Dior, Malick Faye, Ramatoulaye Diagne Gueye, Aminata Diack Mbaye (CH Albert Royer).

Togo, Lomé:

Adults: Akessiwe Patassi*, Awèrou Kotosso, Benjamin Goilibe Kariyare, Gafarou Gbadamassi, Agbo Komi, Kankoé Edem Mensah-Zukong, Pinuwe Pakpame (CHU Tokoin/Sylvanus Olympio).

Pediatrics: Koko Lawson-Evi*, Yawo Atakouma, Elom Takassi, Améyo Djeha, Ayoko Ephoévi-gah, Sherifa El-Hadj Djibril (CHU Tokoin/Sylvanus Olympio).

**Operational and Statistical Team:** Jean-Claude Azani (Abidjan, Côte d'Ivoire), Eric Balestre (Bordeaux, France), Serge Bessekon (Abidjan, Côte d'Ivoire), Sophie Karcher (Bordeaux, France), Jules Mahan Gonsan (Abidjan, Côte d'Ivoire), Jérôme Le Carrou (Bordeaux, France), Séverin Lenaud (Abidjan, Côte d'Ivoire), Célestin Nchot (Abidjan, Côte d'Ivoire), Karen Malateste (Bordeaux, France), Amon Roseamonde Yao (Abidjan, Côte d'Ivoire).

**Administrative Team:** Madikona Dosso (Abidjan, Côte d'Ivoire), Alexandra Doring (Bordeaux, France), Adrienne Kouakou (Abidjan, Côte d'Ivoire), Elodie Rabourdin (Bordeaux, France), Jean Rivenc (Pessac, France).

**Consultants/ Working Groups:** Xavier Anglaret (Bordeaux, France), Boubacar Ba (Bamako, Mali), Renaud Becquet (Bordeaux, France), Juan Burgos Soto (Bordeaux, France), Jean Bosco Essanin (Abidjan), Andrea Ciaranello (Boston, USA), Sébastien Datté (Abidjan, Côte d'Ivoire), Sophie Desmonde (Bordeaux, France), Jean-Serge Elvis Diby (Abidjan, Côte d'Ivoire), Geoffrey S. Gottlieb* (Seattle, USA), Apollinaire Gninlgninrin Horo (Abidjan, Côte d'Ivoire), Julie Jesson (Bordeaux, France), Serge N'zoré Kangah (Abidjan, Côte d'Ivoire), David Meless (Abidjan, Côte d'Ivoire), Aida Mounkaila-Harouna (Bordeaux, France), Camille Ndondoki (Bordeaux, France), Caroline Shiboski (San Francisco, USA), Boris Tchounga (Abidjan, Côte d'Ivoire), Rodolphe Thiébaut (Bordeaux, France), Gilles Wandeler (Dakar, Senegal).

**Website:** http://www.mereva.net/iedea

## References

[1] CASCADE, Collaboration (2003). Determinants of survival following HIV-1 seroconversion after the introduction of HAART. Lancet.

[2] The Antiretroviral Therapy in Lower Income Countries (ART-LINC) Collaboration (2006). The Antiretroviral Therapy (ART) Cohort Collaboration. Mortality of HIV-1-infected patients in the first year of antiretroviral therapy: comparison between low-income and high-income countries. Lancet.

[3] Laurent C, Diakhate N, Gueye NFN, Toure MA, Sow PS, Faye MA (2002). The Senegalese government's highly active antiretroviral therapy initiative: an 18-month follow-up study. AIDS.

[4] Seyler C, Messou E, Gabillard D, Inwoley A, Alioum A, Anglaret X (2007). Morbidity before and after HAART initiation in Sub-Saharan African HIV-infected adults: a recurrent event analysis. AIDS Res Hum Retroviruses.

[5] UNAIDS (2013). Global report. UNAIDS Report on the global AIDS epidemic2013 [Internet].

[6] UNAIDS (2012). UNAIDS Report on the global AIDS epidemic [Internet]. Global report.

[7] Eholie SP, Aoussi FE, Ouattara IS, Bissagnene E, Anglaret X (2012). HIV treatment and care in resource-constrained environments: challenges for the next decade. J Int AIDS Soc.

[8] Moh R, Danel C, Messou E, Ouassa T, Gabillard D, Anzian A (2007). Incidence and determinants of mortality and morbidity following early antiretroviral therapy initiation in HIV-infected adults in West Africa. AIDS.

[9] Etard JF, Ndiaye I, Thierry-Mieg M, Gueye NF, Gueye PM, Laniece I (2006). Mortality and causes of death in adults receiving highly active antiretroviral therapy in Senegal: a 7-year cohort study. AIDS.

[10] Lawn SD, Myer L, Orrell C, Bekker LG, Wood R (2005). Early mortality among adults accessing a community-based antiretroviral service in South Africa: implications for programme design. AIDS.

[11] Gabillard D, Lewden C, Ndoye I, Moh R, Segeral O, Tonwe-Gold B (2013). Mortality, AIDS-morbidity and loss to follow-up by current CD4 cell count among HIV-1 infected adults receiving antiretroviral therapy in Africa and Asia: data from the ANRS 12222 collaboration. J Acquir Immune Defic Syndr.

[12] Lawn SD, Badri M, Wood R (2005). Tuberculosis among HIV-infected patients receiving HAART: long term incidence and risk factors in a South African cohort. AIDS.

[13] Curtis AJ, Marshall CS, Spelman T, Greig J, Elliot JH, Shanks L (2012). Incidence of WHO stage 3 and 4 conditions following initiation of anti-retroviral therapy in resource limited settings. PLoS One.

[14] Beauliere A, Toure S, Alexandre PK, Kone K, Pouhe A, Kouadio B (2010). The financial burden of morbidity in HIV-infected adults on antiretroviral therapy in Cote d'Ivoire. PLoS One.

[15] Boyer S, Marcellin F, Ongolo-Zogo P, Abega SC, Nantchouang R, Spire B (2009). Financial barriers to HIV treatment in Yaounde, Cameroon: first results of a national cross-sectional survey. Bull World Health Organ.

[16] Boulle A, Orrel C, Kaplan R, Van Cutsem G, McNally M, Hilderbrand K (2007). Substitutions due to antiretroviral toxicity or contraindication in the first 3 years of antiretroviral therapy in a large South African cohort. Antivir Ther.

[17] Lewden C, May T, Rosenthal E, Burty C, Bonnet F, Costagliola D (2008). Changes in causes of death among adults infected by the Human Immunodeficiency Virus (HIV) between 2000 and 2005. The “Mortalité 2000 & 2005” surveys (ANRS EN19 and Mortavic). J Acquir Immune Defic Syndr.

[18] Teja VD, Sudha T, Lakshmi V (2007). Causes and pattern of mortality in HIV-infected, hospitalized patients in a tertiary care hospital: a fourteen year study. Indian J Med Sci.

[19] Kumar A, Kilaru KR, Sandiford S, Forde S (2007). Profile of hospitalized HIV-infected persons in the highly active antiretroviral therapy era in Barbados. J Int Assoc Physicians AIDS Care (Chic Ill).

[20] Saleri N, Capone S, Pietra V, De Iaco G, Del Punta V, Rizzi M (2009). Outcome and predictive factors of mortality in hospitalized HIV-patients in Burkina Faso. Infection.

[21] Agaba PA, Digin E, Makai R, Apena L, Agbaji OO, Idoko JA (2011). Clinical characteristics and predictors of mortality in hospitalized HIV-infected Nigerians. J Infect Dev Ctries.

[22] Kra O, Aba YT, Yao KH, Ouattara B, Abouo F, Tanon KA (2013). Clinical, biological, therapeutic and evolving profile of patients with HIV infection hospitalized at infectious and tropical diseases unit in Abidjan (Ivory Coast). Bull Soc Pathol Exot.

[23] World Health Organization (2007). WHO case definitions of HIV for surveillance and revised clinical staging and immunological classification of HIV-related disease in adults and children [Internet]. http://www.who.int/hiv/pub/guidelines/hivstaging/en/index.html.

[24] Anglaret X, Dakoury-Dogbo N, Bonard D, Toure S, Combe P, Ouassa T (2002). Causes and empirical treatment of fever in HIV-infected adult outpatients, Abidjan, Cote d'Ivoire. AIDS.

[25] Anglaret X, Messou E, Oaussa T, Toure S, Dakoury-Dogbo N, Combe P (2003). Pattern of bacterial disease in a cohort of HIV-1 infected adults receiving cotrimoxazole prophylaxis, Abidjan, Côte d'Ivoire. AIDS.

[26] Shelburne SA, Montes M, Hamill RJ (2006). Immune reconstitution inflammatory syndrome: more answers, more questions. J Antimicrob Chemother.

[27] MacLennan R, IARC (1991). Items of patient information which may be collected by registries. Cancer registration: principles and methods.

[28] The Uppsala Monitoring Centre (2012). The use of the WHO-UMC system for standardised case causality assessment [Internet]. http://who-umc.org/Graphics/26649.pdf.

[29] World Health Organization (1993). International classification of diseases.

[30] Smith de Cherif TK, Schoeman JH, Cleary S, Meintjes G, Rebe K, Maartens G (2009). Early severe morbidity and resource utilization in South African adults on antiretroviral therapy. BMC Infect Dis.

[31] Iwuji C, Mayanja B, Weiss H, Atuhumuza E, Hughes P, Maher D (2011). Morbidity in HIV-1-infected individuals before and after the introduction of antiretroviral therapy: a longitudinal study of a population-based cohort in Uganda. HIV Med.

[32] The IeDEA, ART Cohort Collaborations (2014). Immunodeficiency at the start of combination antiretroviral therapy in low-, middle-, and high-income countries. J Acquir Immune Defic Syndr.

[33] Fortes Deguenonvo L, Manga NM, Diop SA, Dia Badiane NM, Seydi M, Ndour CT (2011). Current profile of HIV-infected patients hospitalized in Dakar (Senegal) [French]. Bull Soc Pathol Exot.

[34] Oliveira MT, Latorre Mdo R, Greco DB (2012). The impact of late diagnosis on the survival of patients following their first AIDS-related hospitalization in Belo Horizonte, Brazil. AIDS Care.

[35] Lawn SD, Little F, Bekker LG, Kaplan R, Campbel E, Orrell C (2009). Changing mortality risk associated with CD4 cell response to antiretroviral therapy in South Africa. AIDS.

[36] Cox JA, Lukande RL, Lucas S, Nelson AM, Van Marck E, Colebunders R (2010). Autopsy causes of death in HIV-positive individuals in sub-Saharan Africa and correlation with clinical diagnoses. AIDS Rev.

[37] Crump JA, Ramadhani HO, Morrissey AB, Saganda W, Mwako MS, Yang LY (2011). Invasive bacterial and fungal infections among hospitalized HIV-infected and HIV-uninfected adults and adolescents in northern Tanzania. Clin Infect Dis.

[38] Desalu OO, Oluwafemi JA, Ojo O (2009). Respiratory diseases morbidity and mortality among adults attending a tertiary hospital in Nigeria. J Bras Pneumol.

[39] Anglaret X, Minga A, Gabillard D, Ouassa T, Messou E, Morris B (2012). AIDS and non-AIDS morbidity and mortality across the spectrum of CD4 cell counts in HIV-infected adults before starting antiretroviral therapy in Cote d'Ivoire. Clin Infect Dis.

[40] Martinson NA, Karstaedt A, Venter WD, Omar T, King P, Mbengo T (2007). Causes of death in hospitalized adults with a premortem diagnosis of tuberculosis: an autopsy study. AIDS.

[41] Kouanda S, Meda IB, Nikiema L, Tiendrebeogo S, Doulougou B, Kabore I (2012). Determinants and causes of mortality in HIV-infected patients receiving antiretroviral therapy in Burkina Faso: a five-year retrospective cohort study. AIDS Care.

[42] Cohen T, Murray M, Wallengren K, Alvarez GG, Samuel EY, Wilson D (2012). The prevalence and drug sensitivity of tuberculosis among patients dying in hospital in KwaZulu-Natal, South Africa: a postmortem study. PLoS Med.

[43] Lawn SD, Harries AD, Meintjes G, Getahun H, Havlir DV, Wood R (2012). Reducing deaths from tuberculosis in antiretroviral treatment programmes in sub-Saharan Africa. AIDS.

[44] Kambugu A, Meya DB, Rhein J, O'Brien M, Janoff EN, Ronald AR (2008). Outcomes of cryptococcal meningitis in Uganda before and after the availability of highly active antiretroviral therapy. Clin Infect Dis.

[45] Jarvis JN, Meintjes G, Harrison TS (2010). Outcomes of cryptococcal meningitis in antiretroviral naive and experienced patients in South Africa. J Infect.

[46] Aoussi EF, Ehui E, Dembele JP, Kolia-Diafouka P, Elloh NF, Ouattara SI (2012). Cryptoccocal meningitis and HIV in the era of HAART in Cote d'Ivoire. Med Mal Infect.

[47] World Health Organization (2011). Rapid advice: diagnosis, prevention and management of cryptococcal disease in HIV-infected adults, adolescents and children [Internet].

[48] Jarvis JN, Percival A, Bauman S, Pelfrey J, Meintjes G, Williams GN (2011). Evaluation of a novel point-of-care cryptococcal antigen test on serum, plasma, and urine from patients with HIV-associated cryptococcal meningitis. Clin Infect Dis.

[49] Marshall CS, Curtis AJ, Spelman T, O'Brien DP, Greig J, Shanks L (2013). Impact of HIV-associated conditions on mortality in people commencing anti-retroviral therapy in resource limited settings. PLoS One.

[50] Whitworth J, Morgan D, Quigley M, Smith A, Mayanja B, Eotu H (2000). Effect of HIV-1 and increasing immunosuppression on malaria parasitaemia and clinical episodes in adults in rural Uganda: a cohort study. Lancet.

[51] Srikanth BA, Babu SC, Yadav HN, Jain SK (2012). Incidence of adverse drug reactions in human immune deficiency virus-positive patients using highly active antiretroviral therapy. J Adv Pharm Technol Res.

[52] Buchacz K, Baker RK, Moorman AC, Richardson JT, Wood KC, Holmberg SD (2008). Rates of hospitalizations and associated diagnoses in a large multisite cohort of HIV patients in the United States, 1994–2005. AIDS.

[53] Berry SA, Fleishman JA, Moore RD, Gebo KA (2012). Trends in reasons for hospitalization in a multisite United States cohort of persons living with HIV, 2001–2008. J Acquir Immune Defic Syndr.

[54] Lewden C, Bouteloup V, De Wit S, Sabin C, Mocroft A, Wasmuth JC (2012). All-cause mortality in treated HIV-infected adults with CD4 >=500/mm3 compared with the general population: evidence from a large European observational cohort collaboration. Int J Epidemiol.

